# Gibberellins promote nodule organogenesis but inhibit the infection stages of nodulation

**DOI:** 10.1093/jxb/ery046

**Published:** 2018-02-08

**Authors:** Erin L McAdam, James B Reid, Eloise Foo

**Affiliations:** School of Biological Sciences, University of Tasmania, Hobart, Tasmania, Australia

**Keywords:** DELLA proteins, ethylene, gibberellin, infection thread, nitrogen fixation, nodulation, nodule organogenesis, pea

## Abstract

Leguminous plant roots can form a symbiosis with soil-dwelling nitrogen-fixing rhizobia, leading to the formation of a new root organ, the nodule. Successful nodulation requires co-ordination of spatially separated events in the root, including infection in the root epidermis and nodule organogenesis deep in the root cortex. We show that the hormone gibberellin plays distinct roles in these epidermal and cortical programmes. We employed a unique set of genetic material in pea that includes severely gibberellin-deficient lines and *della*-deficient lines that enabled us to characterize all stages of infection and nodule development. We confirmed that gibberellin suppresses infection thread formation and show that it also promotes nodule organogenesis into nitrogen-fixing organs. In both cases, this is achieved through the action of DELLA proteins. This study therefore provides a mechanism to explain how both low and high gibberellin signalling can result in reduced nodule number and reveals a clear role for gibberellin in the maturation of nodules into nitrogen-fixing organs. We also demonstrate that gibberellin acts independently of ethylene in promoting nodule development.

## Teaser

Gibberellin plays a dual role in legumes during uptake and accommodation of nitrogen-fixing bacteria; inhibiting infection at the epidermis but promoting nodule organogenesis and ultimate function.

## Introduction

Nodulation is the interaction between compatible legumes and rhizobial bacteria that involves the uptake of these soil bacteria into newly formed root organs termed nodules. The plant strictly controls the extent of nodulation, by various means such as acting through plant hormones including the gibberellins (GAs) (Ferguson and Mathesius, 2014; Foo, 2017). GAs are diterpenoids, and the active forms of GA are perceived by the GID1 (GA insensitive dwarf1) receptor that in turn binds to and activates the degradation of DELLA proteins, transcriptional regulators that repress GA responses (Claeys *et al.*, 2014). High GA levels reduce DELLA protein levels and thus alleviate the repression of GA responses.

Early literature examining the role of GAs in nodulation used both application and genetic approaches, and reported both negative and positive effects of GAs on the number of nodules formed (for a review, see Hayashi *et al.*, 2014). In particular, studies with a range of GA biosynthesis mutants in pea, *na* (*nana*), *ls*, and *lh*, revealed that low GA signalling can suppress nodule formation (Ferguson *et al.*, 2005). The small number of nodules that do form on severely GA-deficient *na* mutants are small and white, with reduced meristem size (Ferguson *et al.*, 2005, 2011), suggesting that GA may be required to promote nodule organogenesis. However, overproduction of bioactive GA in the *sln* (*slender*) catabolism mutant (Lester *et al.*, 1999) can reduce nodulation (Ferguson *et al.*, 2005). Likewise, *della* mutants in pea (*la cry-s*; Weston *et al.*, 2008) that display constitutive GA responses also have a reduced number of nodules compared with wild-type plants (Ferguson *et al.*, 2011), suggesting a complex role for GA signalling as both a promoter and inhibitor of nodulation. This requirement for an optimal level of GA signalling for nodule formation and development in pea was confirmed by studies using application to wild-type and GA-deficient plants (Ferguson *et al.*, 2005). This is consistent with results in *Sesbania rostrata* as, during crack entry, application of a GA biosynthesis inhibitor before bacterial inoculation promoted outer cortical colonization and bacterial accumulation in infection pockets, but suppressed all nodule organogenesis (Lievens *et al.*, 2005).

Nodulation at the whole-root level consists of at least two spatially separate programmes, infection at the epidermis and nodule organogenesis originating in the inner cortex. Central to these events is the perception of specific rhizobial-produced Nod lipochito-oligosaccharides (Nod factors) by receptor-like kinases (NFP and LYK3) that in turn activate DMI2 (Does not Make Infections2) and DMI1. This is sensed by DMI3, that along with parallel pathways influences a range of transcription factors (e.g. NIN, NSP1, NSP2, and IPD3) that in turn co-ordinate the expression of nodulation-associated genes (Venkateshwaran *et al.*, 2013; Genre and Russo, 2016). This pathway induces physical changes that enable colonization, including root hair curling, infection thread formation, and cell division in the inner cortical and pericycle cell layers, leading to nodules that, depending on the plant species, may either retain a meristem at maturity (indeterminate) or not (determinate) (Ferguson *et al.*, 2010). Subsequent nodule formation is tightly regulated by a systemic control system termed the autoregulation of nodulation pathway (Soyano *et al.*, 2013).

Recent studies in *Lotus japonicus* and *Medicago truncatula* have examined the role of elevated GA signalling via the DELLA proteins in nodulation, particularly the responses at the epidermis. In both species, disrupted DELLA function, and hence constitutive GA responses, resulted in reduced nodule numbers (Maekawa *et al.*, 2009; Fonouni-Farde *et al.*, 2016; Jin *et al.*, 2016). GA application to wild-type plants in these species also suppressed nodule number, and a small positive effect on nodule number was observed after application of GA biosynthesis inhibitors (Maekawa *et al.*, 2009; Fonouni-Farde *et al.*, 2016; Jin *et al.*, 2016). These results have led to hypotheses suggesting that GAs, acting through DELLAs, suppress nodulation (Fonouni-Farde *et al.*, 2016; Jin *et al.*, 2016). Further, they provide molecular and physical evidence that GA inhibits nodulation events occurring at the epidermis and/or early in the nodulation process such as infection thread formation (Lievens *et al.*, 2005; Maekawa *et al.*, 2009; Fonouni-Farde *et al.*, 2016). Consistent with this, Nod factor activated expression of transcription factors genes such as *NSP1*, *NSP2*, and *NIN*, and downstream early nodulation (*ENOD*) genes are suppressed by co-treatment or pre-treatment with GA in wild-type *Lotus* and *Medicago* and, in the absence of GA treatment, in *Medicago della* mutant lines (Maekawa *et al.*, 2009; Fonouni-Farde *et al.*, 2016; Jin *et al.*, 2016). Indeed, yeast two-hybrid studies using MtDELLA proteins have informed models that suggest that DELLA may interact with various partners *in vitro*, including NF-YA1, NSP1, NSP2, and IPD3, and DELLAs appear to increase the phosphorylation state of IPD3 *in vitro* (Fonouni-Farde *et al.*, 2016; Jin *et al.*, 2016). It is important to note that the expression pattern of many nodulation-associated genes is complex, with some genes such as *ENOD40*, *ERN1* (*Required for Nodulation1*), *NIN*, and *YC* expressed both early in infection and also later in developing nodules (e.g. Matvienko *et al.*, 1994; Borisov *et al.*, 2003; Middleton *et al.*, 2007; Zanetti *et al.*, 2010), while other genes are only expressed in developing nodule primordia and/or maturing nodules, such as *YA1/HAP2*, *YB*, and *VAP* (Combier *et al.*, 2006; Murray *et al.*, 2011; Soyano *et al.*, 2013). Studies examining the influence of GA level and/or signalling on the expression of these genes during different stages of infection and nodule development are required.

The recent studies that have focused primarily on events at the epidermis cannot explain the clear positive role that GAs play in nodule number and development in several species such as pea and *S. rostrata* outlined above (Ferguson *et al.*, 2005, 2011; Lievens *et al.*, 2005). An untested model explaining these conflicting results is that GAs suppress early events at the epidermis, such as infection thread formation, but promote nodule organogenesis occurring in the cortex. This model is consistent with the negative role of GAs in epidermal events seen across species and the underdeveloped, white nodules in severely GA-deficient pea *na* mutants outlined above. Indeed, GA is well known for playing a positive role in cell division, differentiation, and organogenesis (Claeys *et al.*, 2014). This model is also consistent with the fact that *della* mutants in several species can form mature pink nodules (Ferguson *et al.*, 2005; Jin *et al.*, 2016), indicating that constitutive GA signalling does not suppress nodule organogenesis.

Here, we use pea to test this model. Pea is unique, being the only legume species with both GA-deficient and DELLA-deficient mutants, and lines combining both, allowing the examination of the effects of both GA levels and signalling on the full range of processes from infection through to mature nodules. This range of mutants is essential as DELLAs have been shown to interact with several other hormone signalling pathways (Davière and Achard, 2016), meaning previous studies in *Medicago* and *Lotus* employing *della* mutants and/or overexpressing lines do not allow us to determine whether the effects observed are due solely to the action of GA signalling through DELLA proteins. It is also not clear from these studies, which primarily focus on early epidermal events, whether DELLAs positively or negatively influence nodule formation and/or function. We examine each stage of nodule development in *na-1* plants that exhibit severe GA deficiency due to a null mutation in the key GA biosynthesis enzyme *ent*-kaurenoic acid oxidase (Davidson *et al.*, 2003), mutants deficient in DELLA proteins, which act as negative regulators of GA signalling (Weston *et al.*, 2008), and in lines that combine both. We delineate two very different roles for GA, acting as a suppressor of early epidermal events and as a promoter of nodule organogenesis and hence the ability to develop into nitrogen-fixing organs. Ethylene is another key hormone known to regulate nodulation, including by interacting with GA (e.g. Foo *et al.*, 2016*b*), and therefore we also examine the interaction between GA and ethylene at various stages of nodulation using a genetic approach.

## Materials and methods

### Plant material and growth conditions

The *Pisum sativum* L. lines used were the severely GA-deficient *na-1* (Davidson *et al.*, 2003) derived from wild-type WL1769 (Reid *et al.*, 1983) and crossed onto a cv. Torsdag background where comparisons with other genotypes on this background were required (Foo *et al.*, 2013); the DELLA mutant *NA la cry-s* (Weston *et al.*, 2008); the ethylene-insensitive *ein2* mutant (Weller *et al.*, 2015) derived from cv. Torsdag; the symbiosis mutant *dmi2* (*sym19*; Stracke *et al.*, 2002) derived from cv. Frisson; and the super-nodulating autoregulation of nodulation mutant *nark* (P88; *sym29*; Sagan and Duc, 1996; Krusell *et al.* 2002) derived from cv. Frisson. The triple mutant *na-1 la cry-s* was derived from a cross between cv. Torsdag and Hobart line 188 as described by Foo *et al.* (2013); the double mutant *na-1 ein2* was derived as described by Foo *et al.* (2016*b*); the double mutant *na-1 nark* was derived from a cross between *na* and *nark* (Ferguson *et al.*, 2011). Comparisons were made between the mutant line and the appropriate progenitor line or isogenic wild-type line as described above. *Lupinus angustifolius* (blue lupin) seeds were sourced from a local seed supplier (Mitre 10 Pty Ltd, Hobart, Australia). Seeds were surface sterilized with 70% ethanol and grown in growth cabinets [18 h photoperiod, 20 °C day, 15 °C night, under cool-white fluorescent tubes (100 µmol m^–2^ s^–1^)], two per 14 cm pot in vermiculite, under conditions to exclude rhizobial bacteria as outlined in McAdam *et al.* (2017), unless otherwise stated.

### Infection thread, bacterial accumulation, and developing nodule studies

Seedlings were inoculated 10 d after planting as described by McAdam *et al.* (2017). Briefly, plants were inoculated with *Rhizobium leguminosarum* bv. *viciae* (RLV3841) carrying pXLGD4 (carrying the *lacZ* reporter gene; supplied by John Innes Centre, UK). Nine days after inoculation, secondary roots were harvested and stained using 5-bromo-4-chloro-3-indolyl-β-d-galactopyranoside (X-gal). Root segments were viewed with a Zeiss Axiolab light microscope (Göttingen, Germany) with a ×10 or ×20 objective, and images were taken with a Nikon Digital Sight DS-Fi2 camera (Melville, NY, USA). Measurements were made of the root length, number of blue-stained infection threads, number of blue-stained bacterial accumulations, and number of nodules (at all developmental stages visible with blue stain under a ×20 objective). As *na* mutants have much shorter epidermal cells, infection structures are expressed on a per lateral epidermal cell basis, which was calculated by dividing the root length by the average epidermal cell length. The average epidermal cell length was calculated for each sample from 20 cells, by clearing one root segment from each sample in 5% (v/v) KOH, and taking four images at intervals along the root, excluding the root tip and root elongation zone. The length of five cells from each of the four images was measured using Image J version 1.48 (National Institutes of Health, Bethesda, MD, USA).

### Root hair curling analysis

Plants were treated 10 d after planting, with either 75 ml of a 10% solution from a 3-day-old culture of *R. leguminosarum* bv. *viciae* (RLV248) grown in yeast-mannitol broth or 75 ml of a 10% solution of sterile yeast-mannitol broth (control). At 26 d after treatment, 1–3 secondary roots from 6–9 plants were stained briefly with toluidine blue and examined under a light microscope, and the percentage of curled root hairs was recorded.

### Acetylene reductase nodule function studies

For the acetylene reduction assay, plants were grown for 7 d and inoculated with *R. leguminosarum* bv. *viciae* (RLV248) as previously described (Foo and Davies, 2011). At 4–5 weeks after inoculation, 1.5 g of nodulated root tissue was placed in 136 ml bottles sealed with a gas-tight lid fitted with a septum, with four replicate bottles per genotype. A 0.8 ml aliquot of acetylene was added to each bottle to make a final concentration of 5882.4 ppm, and the roots were incubated for 2.5 h at room temperature. A 0.5 ml sample of gas was taken from each sample with a gas-tight syringe for analysis. The amount of ethylene generated from the reduction of acetylene via nitrogenase was measured by GC-flame ionization detection (FID), with a Varian 450-GC gas chromatograph (Agilent Technologies, USA) equipped with a flame ionization detector and a GS-Q GC 30 m×0.53 mm column (Agilent Technologies). The injector temperature was 70 °C, the oven temperature was 80 °C, and the detector temperature was 120 °C. Hydrogen carrier gas flow was 30.5 ml min^–1^. After analysis, the FW of nodules was measured and ethylene evolved was expressed on a per g (FW) of nodules basis.

### Gene expression studies

For Supplementary Figs S3 and S4 at *JXB* online, plants were grown under sterile conditions for 10 d and for Supplementary Fig. S3 root tip tissue (2–3 plants per replicate) was harvested before inoculation (day 0). The remaining plants were inoculated with 75 ml of a 10% solution of a 3-day-old culture of RLV248, and 2 d and 4 d later root tip tissue was harvested. For Supplementary Fig. S4A, plants were grown in the same way but harvested 12 h and 24 h following inoculation. For Supplementary Figs S5–S7, plants were grown and inoculated as described by Foo and Davies (2011) and 4–5 weeks later individual nodules (including the root segment to which the nodules were attached) were excised. For all gene expression studies, each replicate contained tissue from 2–3 plants.

Tissue was ground and RNA was extracted from ~100 mg of tissue using the ISOLATE II RNA Mini Kit (Bioline, Alexandria, Australia). cDNA was synthesized from 1 µg of RNA using the SensiFAST™ cDNA Synthesis Kit (Bioline). cDNA was diluted, and duplicate, real-time PCRs were performed in a Rotor Gene 2000 (Corbett, USA) using the SensiFAST™ SYBR^®^ Hi-ROX Kit (Bioline) and 100–200 pmol of a primer pair. New primer pairs for genes analysed in this study are given in Supplementary Table S1, and previously published primer pairs are as follows: *PsGA2-ox-2*, *PsGA3-ox*, and *PsGA20-ox* (Foo *et al.*, 2006), *PsENOD12a* and *PsENOD40* (Foo *et al.*, 2016*a*), and the housekeeping gene *PsACTIN* (Foo *et al.*, 2005). Standard curves were created for each gene using serially diluted plasmids containing cloned fragments of each amplicon, and plasmids were sequenced to verify specificity. The average concentration of technical replicates was calculated. The relative gene expression for each biological replicates was determined by calculating the ratio of the gene of interest to the housekeeping gene (*PsACTIN* or *PsTFIIa*) for that sample.

### Hormone application studies

**Fig. 1.**
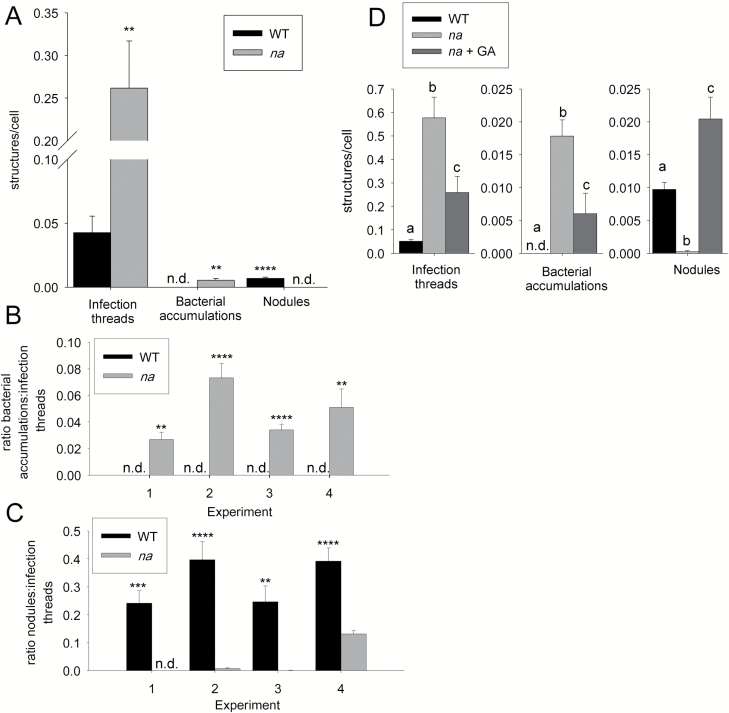
Infection and nodule development in wild type (WT) and gibberellin-deficient *na-1* plants 9 d after inoculation with *lacZ*-labelled *Rhizobium leguminosarum* bv. *viciae.* (A) Number of infection structures, including infection threads, bacterial accumulations, and nodules per root epidermal cell, in experiment 1. (B, C) Data from four independent experiments shown: (B) ratio of bacterial accumulations to infection threads and (C) ratio of nodules to infection threads. (D) Number of infection structures per root epidermal cell in the WT and *na* mutants with and without exogenous gibberellin (10 µg GA_3_). Values are the mean ±SE (1–6 secondary roots from *n*=5–12 plants). For (A–C) asterisks indicate significant differences (**P<0.01, ***P<0.001, ****P<0.0001) *na* was compared with the corresponding WT line and for (D) values with different letters are significantly different (*P*<0.05); n.d., not detected.

For Figs 1D, 5A, and B, pea seeds were nicked and treated at the time of planting with either 5 µl of ethanol (control) or 10 µg of GA_3_ (Merck, Australia) in 5 µl of ethanol. Seeds were planted and inoculated at 7 d as described by Foo and Davies (2011). At 25 d, 5 µl of ethanol (control) or GA_3_ in 5 µl of ethanol was applied to the uppermost mature leaf. For Fig. 1D, roots were stained for *lacZ* and infection structures recorded as described above. For Fig. 5A and B, nodule number and size were recorded as described by Foo *et al.* (2016*b*) at 37 d. For Fig. 5C and D, lupin seeds were nicked and treated at the time of planting with either 10 µl of ethanol (control), 10 µg of GA_3_, and/or 10 µg of paclobutrazol (PAC; Sigma Aldrich Pty. Ltd, Australia) in 10 µl of ethanol, planted and inoculated with appropriate bacteria as described by Foo *et al.*, (2016*a*), and nodule size was recorded at 28 d as described by Foo *et al.* (2016*b*).

### Hormone analysis

For Supplementary Fig. 4A, plants were grown and inoculated as described for gene expression studies, and ~0.5 g FW of root tissue from the infection zone (1 cm of tissue, 1 cm back from root tip, 4–6 plants per replicate) was harvested. For Supplementary Fig. S4B, plants were grown and inoculated as described for gene expression studies and, 12 d after inoculation, 1–2 g FW of whole roots were harvested (2–3 plants per replicate). GA_1_ standard (^2^H_2_-GA_1_) was added to each sample and GA_1_ was extracted and quantified as described by Boden *et al.* (2014) and derivatized as described by Li *et al.* (2016) with the modification as follows; samples were derivitized in 15 µl of 200mM *N*-(3-dimethylaminopropyl)-*N*'-ethylcarbodiimide hydrochloride (EDC; Sigma, Australia) in water (pH 4.5) at 40 °C overnight.

### Transmission electron microscopy

Plants were grown as described for infection thread experiments. Large wild-type nodules (3–4 mm), the largest *na-1* nodules (1–2 mm), and *na-1* roots containing bacterial accumulations were fixed and stored in 2.5% glutaraldehyde in buffer (100 mM sodium phosphate at pH 7, 10 mM KCl, and 1 mM MgCl_2_·6H_2_O), washed twice in 0.1 M PIPES (Sigma Australia) buffer (pH 7), treated with 1% osmium tetroxide in 0.1 M PIPES buffer (pH 7) for 2 h, then washed twice and stored in 0.1 M PIPES buffer. Samples were then treated with 5% uranyl acetate (ProSciTech Pty Ltd, Australia) in 50% ethanol for 30 min, dehydrated through graded ethanol and propylene oxide, and embedded in Procure 812 resin (ProSciTech Pty Ltd) according to the manufacturer’s instructions. Sections 70 nm thick were cut with a Reichert Ultracut S Ultramicrotome, and images of cross-sections captured with an Olympus BX50 microscope (Supplementary Fig. S2). The sections were then collected onto copper grids, and stained with uranyl acetate and Reynolds Lead citrate, then imaged with a Hitachi HT7700 transmission electron microscope at 80 kV at ×1000–×15 000 magnification.

### Statistical analysis

For pairwise comparisons, Student’s *t*-tests were performed in Excel. For other experiments, one- or two-way ANOVAs were performed in R version 3.2.2 (R Core Team, Vienna, Austria), followed by Tukey’s HSD post-tests where appropriate. Prior to analysis, Box–Cox tests were used to determine the optimal transformation to remediate deviations from assumptions of the linear regression model.

## Results

### Gibberellins suppress infection thread formation and the conversion of infection threads to nodules, but promote nodule organogenesis

**Fig. 2.**
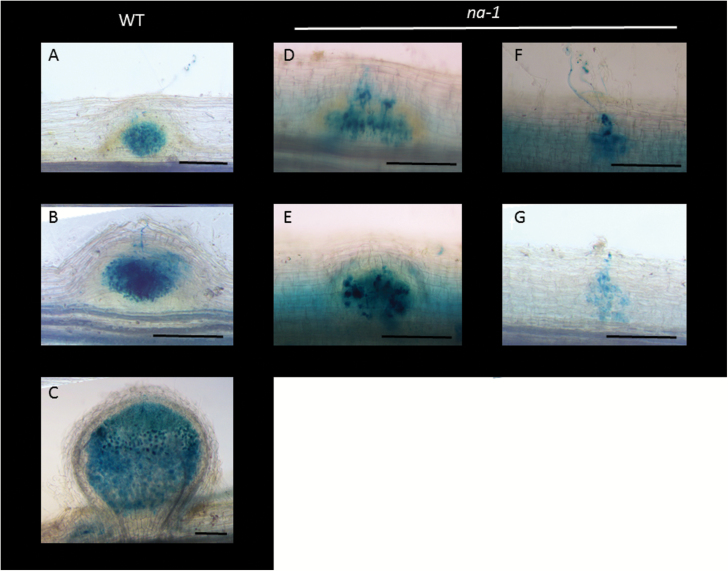
Light microscope images of wild-type (WT) (A–C) and *na-1* mutant roots (D–G) during infection and nodule development 9 d following inoculation with *lacZ*-labelled *Rhizobium leguminosarum* bv. *viciae*. (A, D) Early nodule development, (B, E) later nodule development, (C) mature WT nodule, and (F, G) bacterial accumulations in *na-1* mutant roots. Scale bar=200 μm.

**Fig. 3.**
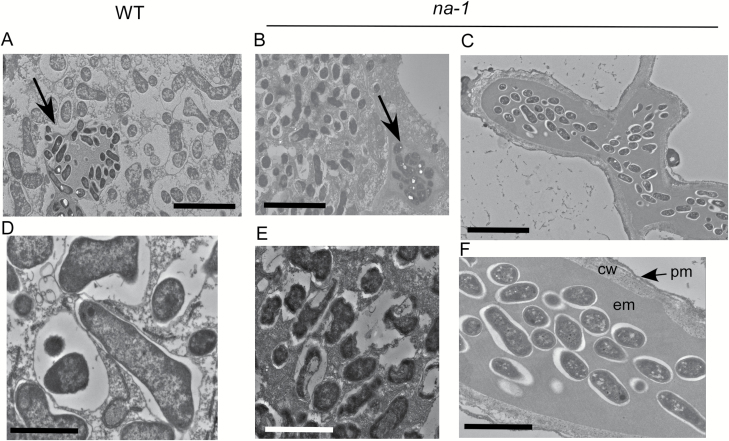
TEM images of wild type (WT) (A, D) and *na-1* mutant (B–F) infection and nodule development 5 weeks following inoculation with *lacZ*-labelled *Rhizobium leguminosarum* bv. *viciae*. (A, B) Central zone of the nodule; the arrow indicates an infection thread. (D, E) Close-up of bacteria inside the central zone. (C) Bacterial accumulation in *na-1* mutant root and (F) close-up of bacterial accumulation, showing bacteria, extracellular matrix (em), plant cell wall (cw), and plasma membrane (pm). For (A–C) scale bar=60 μm, for (D–F) scale bar=20 μm.

It is essential that we reconcile the fact that both high and low GA signalling can result in suppression of the ultimate number of nodules formed in pea (Ferguson *et al.*, 2005, 2011). To pinpoint the influence of GA on the different stages of nodulation (infection and organogenesis), we employed the range of GA mutants available in pea and examined infection using *lacZ*-labelled *R. leguminosarum* bv. *viciae.* Infection was examined in severely GA-deficient *na* mutants compared with its wild-type progenitor (Figs 1–3). It is important to note that *na* roots have significant reductions in cell elongation (Yaxley *et al.*, 2001), so the number of infection events in *na* and wild-type roots was calculated on a per epidermal cell basis. Despite a dramatic reduction in epidermal and cortical cell elongation in *na*, we did not observe any differences in the length of root hairs or the number of root hairs per root in *na* mutants compared with wild-type plants (data not shown).

When bacterial uptake was tracked, there was a striking difference in infection events in GA-deficient *na* mutants compared with the wild type (Figs 1, 2). We found a dramatic increase in the number of infection threads formed in *na* mutants compared with the wild type on a per cell basis (Fig. 1A, *P*<0.01). This is not due to a significant change in the number of root hairs that curled in response to rhizobia, as the frequency of root hair curling was similar in wild-type plants and GA-deficient *na* mutants (Supplementary Fig. S1). However, it does appear that more of the curled root hairs went on to make an infection thread in *na* mutants than occurs in wild-type plants. Despite increased root infection, GA-deficient *na* mutants often formed no nodules (Fig. 1A), although a small number of underdeveloped nodules was observed in some experiments (Figs 1C, 2D, E). This is consistent with previous reports (Ferguson *et al.*, 2005, 2011). Across four independent experiments, the ratio of the number of nodules to infection threads formed in *na* mutants was significantly lower than in wild-type plants (Fig. 1C).

When mature nodules from wild-type plants and the most mature nodules seen in *na* mutants (Fig. 2C and E, respectively) were examined using TEM, there was a striking difference in the morphology of the bacteria inside the central zone. In the wild type, bacteria in this zone had differentiated into large bacteroids, clearly distinct from bacteria inside the infection threads (Fig. 3A). In contrast, bacteria in the central zone of *na* nodules were significantly smaller than those in nodules from wild-type plants, and more closely resembled bacteria inside infection threads (Fig. 3B). Indeed, the bacteria inside nodules from *na* plants appeared to be somewhat deteriorated, often displaying damaged peribacteroid membranes and symbiosome membranes, compared with intact and undamaged bacteroids in wild-type nodules (Fig. 3D, E).

Another striking feature of *na* mutants was that a proportion of infection threads went on to form ramified structures within the root cortex that we have termed bacterial accumulations (Figs 1A, B, 2F, G). These bacterial structures were never observed in wild-type plants (Fig. 1B). Bacterial accumulations are distinct from nodules, as they have none of the associated cell divisions that occur in nodules (Fig. 2F, G; Supplementary Fig. S2). When examined using TEM, the bacterial accumulation resembles an infection thread and the bacteria inside the bacterial accumulations of *na* plants resembled those in infection threads (Fig. 3C, F).

The elevated infection thread number, reduced nodule number, and presence of bacterial accumulations in *na* plants appeared to be due to GA deficiency, as all were significantly reversed by the addition of exogenous GA_3_ to *na* plants (Fig. 1D). There was a significant reduction of infection threads and bacterial accumulations in *na* plants treated with GA_3_ compared with untreated *na* plants, and this was accompanied by a significant increase in nodule numbers on these plants compared with untreated *na* and wild-type plants. Further, a small number of bacterial accumulations could be induced in wild-type plants by treatment with the GA biosynthesis inhibitor PAC (data not shown).

### GAs influence nodulation through DELLAs

**Fig. 4.**
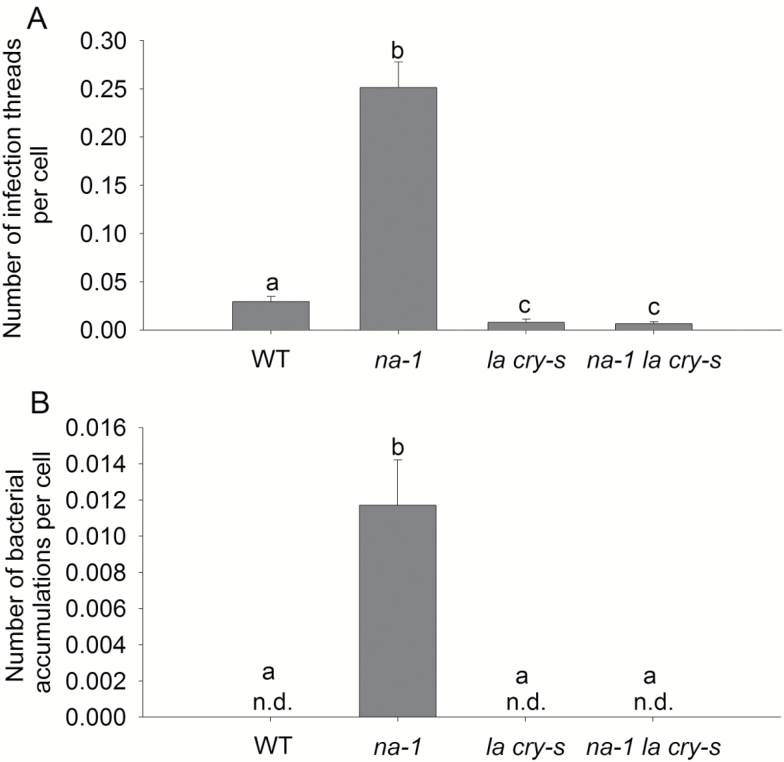
Rhizobial infection in wild-type (WT), gibberellin-deficient mutant *na-1*, the DELLA protein mutant *la cry-s*, and the triple mutant *na-1 la cry-s* plants 5 weeks after inoculation with *lacZ*-labelled *Rhizobium leguminosarum* bv. *viciae.* (A) The number of infection threads per root epidermal cell and (B) the number of bacterial accumulations per root epidermal cell. Values are mean ±SE (one secondary root from *n*=12 plants); n.d., not detected. Values with different letters are significantly different (*P*<0.05).

Similar to previous reports using *Medicago* DELLA-deficient lines (Fonouni-Farde *et al.*, 2016; Jin *et al.*, 2016), infection thread formation was significantly reduced in DELLA-deficient pea *la cry-s* double mutants compared with wild-type plants (Fig. 4A). Importantly, GA-deficient *na* phenotypes rely completely on signalling via DELLA proteins, as the number of infection threads and bacterial accumulations formed in triple mutant GA- and DELLA-deficient *na la cry-s* plants was not significantly different from that in DELLA-deficient *la cry-s* mutants (Fig. 4). This is important, as it provides clear evidence that the previously noted influence of *della* mutants on early nodulation events in other legume species (Fonouni-Farde *et al.*, 2016; Jin *et al.*, 2016) appears to be entirely due to disruption of GA signalling, rather than some other effect of DELLA proteins. As previously reported (Ferguson *et al.*, 2011), *na la cry-s* and *la cry-s* mutants had similar numbers of nodules but both had significantly fewer nodules per g root DW than wild-type plants [wild type 0.010 ± 0.001, *na la cry-s* 0.002 ± 0.00003, *la cry-s* 0.003 ± 0.0009; values are mean ±SE (*n*=12)].

### Early nodulation gene expression and gibberellin biosynthesis following inoculation

Molecular events that occur in the days following inoculation with rhizobia include induction of early nodulation (*ENOD*) genes. In pea, these include *ENOD40*, *ENOD12a*, *ENOD12b*, and *NIN* (e.g. Scheres *et al.*, 1990; Govers *et al.*, 1991; Matvienko *et al.*, 1994; Zhukov *et al.*, 2008). We monitored expression of these genes in root tips of the wild type and GA-deficient *na* mutants 0, 2, and 4 d after inoculation with rhizobia (Supplementary Fig. S3). Small but significant increases in gene expression were observed in wild-type plants in the days following inoculation. Mutant *na* plants displayed contrasting patterns of gene expression, with little induction in the expression of *ENOD40* and *ENOD12b* but significantly higher induction of expression of *ENOD12a* and *NIN* at 4 d after inoculation compared with inoculated wild-type plants.

Studies in several species have reported changes in the expression of GA biosynthesis and catabolism genes in the hours and days following inoculation, suggesting that bioactive GA levels may be elevated (for a review, see Hayashi *et al.*, 2014). However, in wild-type pea plants, we found no significant difference in the expression of key GA biosynthesis genes, *PsGA20ox2* and *PsGA3ox1*, or the GA catabolism gene, *PsGA2ox1*, between inoculated and mock-inoculated plants 12 h and 24 h after challenge (Supplementary Fig. S4A). Indeed, we also found no change in bioactive GA_1_ levels in root tips 24 h and 48 h following inoculation (Supplementary Fig. S4A). Similarly, there was no difference in the expression of these genes or the level of GA_1_ between inoculated wild type and non-nodulating *dmi2* pea mutants (Supplementary Fig. S4B), disrupted in an essential element of the Nod LCO signalling pathway (Stracke *et al.*, 2002).

### Gibberellin promotes nodule organogenesis

**Fig. 5.**
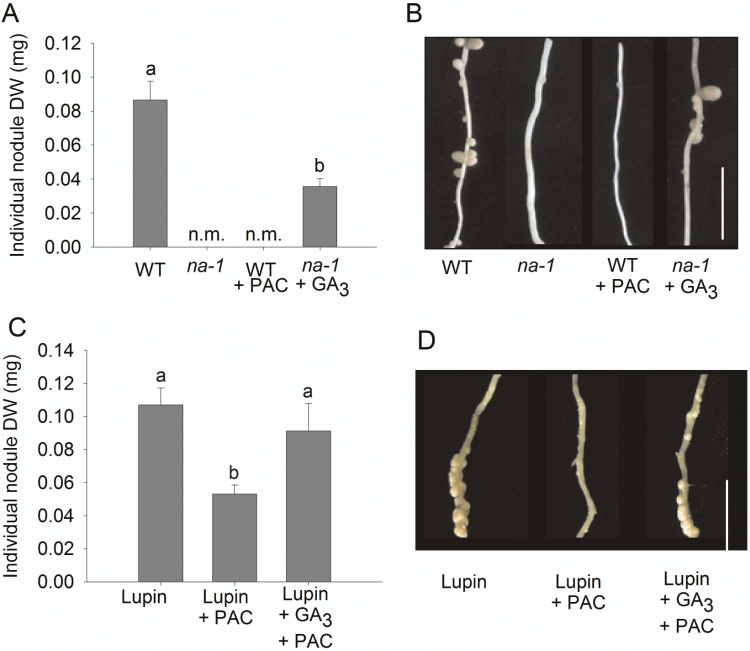
Effect of exogenous gibberellin (GA_3_) on nodule size in pea and lupin 3–4 weeks after inoculation with appropriate bacteria. (A) Average dry weight (DW) of individual nodules of wild-type (WT) and *na-1* pea mutants treated with the GA biosynthesis inhibitor paclobutrazol (PAC) or GA_3_, (B) Photo of nodules on secondary roots of pea. (C) Average DW of individual nodules of lupin treated with PAC or PAC plus GA_3_. (D) Photo of nodules on secondary roots of lupin. (A, C) Values are mean ±SE (*n*=6–10) and values with different letters are significantly different (*P*<0.05). n.m., not measurable. (B, D) Scale bar=2cm.

Accompanying infection at the epidermis is nodule organogenesis. This involves de-differentiation, division, expansion, and re-differentiation of inner cortical cells, into which the bacteria from infection threads enter and ultimately fix nitrogen (Ferguson *et al.*, 2010). It has previously been reported that the few nodules that sometimes form on GA-deficient *na* mutants are always much smaller than those formed on wild-type plants (Ferguson *et al.*, 2005). We found that this suppression of nodule size could be mimicked in wild-type peas by the addition of a high dose of the GA biosynthesis inhibitor PAC and partially rescued in *na* mutants by addition of GA_3_ (Fig. 5A, B; Supplementary Table S2). This positive influence of GA on nodule size was also seen in blue lupin (*Lupinus angustifolious*) plants, with a significant suppression of nodule size in PAC-treated lupin compared with untreated plants that could be rescued by addition of GA_3_ (Fig. 5C, D). This suggests that the positive influence of GA on nodule size occurs in species that form nodules through root hair (pea) or crack entry (lupin), and also species that form indeterminate (pea) or determinate (lupin) nodules.

Nodule organogenesis is accompanied by the expression of many key nodulation genes. In pea we monitored the expression of a range of genes known to be expressed in nodules of pea and/or other legumes (e.g. *NIN*, Borisov *et al.*, 2003; *ENOD40*, Matvienko *et al.*, 1994; *ERN*, Middleton *et al.*, 2007; *YA1/HAP2*, Combier *et al.*, 2006; *YB*, Soyano *et al.*, 2013; *YC*Zanetti *et al.*, 2010; and *VAP*, Murray *et al.*, 2011). To determine the influence of GA deficiency on the expression of these genes, we examined gene expression in sections of mature wild-type and *na* mutant roots bearing nodules (Supplementary Fig. S5). The wild-type nodules were large and pink, while the nodules that did form on these *na* mutants typically had a small, white appearance. The expression of several key genes, *ENOD40*, *YC*, and *VAP*, was significantly lower in the nodules on *na* plants compared with wild-type plants. In contrast, the expression of *NIN* and *YA1* was significantly elevated in *na* nodules compared with wild-type nodules. This influence of the *na* mutation on the expression of these genes required DELLA, as the expression of all of these genes was not significantly different in wild-type, DELLA-deficient *la cry-s*, and triple mutant *na la cry-s* plants (Supplementary Fig. S6). Indeed, the fact that expression of these nodule marker genes was not disrupted at all in DELLA-deficient *la cry-s* and triple mutant GA- and DELLA-deficient *na la cry-s* plants is important, as it suggests that constitutive GA signalling in these mutant lines does not compromise nodule development.

### Gibberellin–ethylene interactions during infection and nodule development

**Fig. 6.**
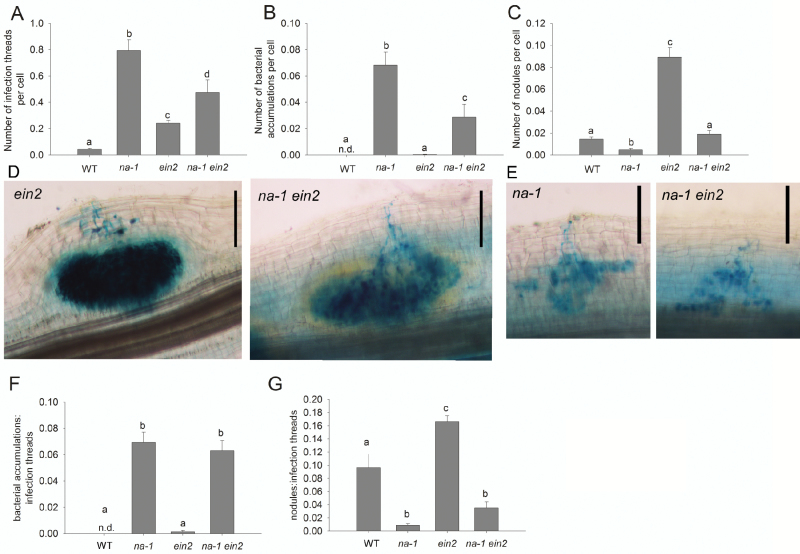
Nodule development in wild-type (WT), gibberellin-deficient mutant *na-1*, ethylene-insensitive mutant *ein2*, and the double mutant *na-1 ein2* plants 4 weeks after inoculation with *lacZ*-labelled *Rhizobium leguminosarum* bv. *viciae.* (A) Number of infection threads per root epidermal cell, (B) number of bacterial accumulations per root epidermal cell, and (C) number of nodules per root epidermal cell. (D) Photos of stained roots of *ein2* and *na-1 ein2* mutant plants showing developing nodules and (E) photos of stained roots of *na-1* and *na-1 ein2* mutant plants showing bacterial accumulations, scale bars=200 µm. (F) Ratio of bacterial accumulations to infection threads and (G) ratio of nodules to infection threads. Values are mean ±SE (three secondary roots measured from *n*=6 plants); n.d., not detected. Values with different letters are significantly different (*P*<0.05).

Previous studies have revealed that GA may interact with ethylene during nodulation in pea. GA-deficient *na* mutants produce more ethylene and this appears to contribute at least in part to the low nodule number in this mutant, as chemical blockers of ethylene synthesis or disruption of ethylene perception, through the *ein2* mutation, could elevate nodule number in *na* (Ferguson *et al.*, 2011; Foo *et al.*, 2016*b*). We investigated this interaction in more detail by examining infection events with *lacZ*-labelled rhizobium in *na ein2* double mutants, single mutant parents, and wild-type plants (Fig. 6). The phenotype of *na* mutants was consistent with previous experiments (Figs 1, 6A–C). Ethylene-insensitive *ein2* parents also displayed elevated infection thread number but also had significantly more nodules than wild-type plants. Mutant *ein2* plants occasionally formed a very small number of bacterial accumulations (two out of six plants had one or two bacterial accumulations across all roots scored, Fig. 6B), although this was not significantly different from the wild type.

The elevated infection thread number in *na* mutants is clearly not due to elevated ethylene as, like both parental lines, GA-deficient and ethylene-insensitive *na ein2* double mutant plants formed significantly more infection threads than the wild type (Fig. 6A). A functioning GA synthesis system appears to suppress infection threads in *ein2* mutants to some extent, as infection thread number was slightly elevated in *na ein2* compared with the *ein2* parent. This suggests that GA and ethylene act relatively independently to suppress infection thread formation in pea. GA also appears to suppress bacterial accumulations relatively independently of ethylene, as, like the *na* parent, *na ein2* double mutants formed significantly more bacterial accumulations than the wild type or *ein2* mutants (Fig. 6B), and these accumulations had a similar appearance to those formed in *na* plants (Fig. 6E). Indeed, the ratio of bacterial accumulations to infection threads was not significantly different between *na* and *na ein2* plants (Fig. 6F).

The number of nodules on *na ein2* plants was significantly elevated compared with the *na* parent and was not significantly different from that seen on wild-type plants (Fig. 6C). This increase in nodule number in *na ein2* plants is consistent with the hypothesis that GA promotes nodule formation in part by suppressing ethylene. This is reflected in the fact that the low ratio of nodules to infection threads seen in GA-deficient *na* mutants is somewhat elevated in an *ein2* background, although this was not significant (Fig. 6G). Overall, it appears that GA suppresses infection thread formation relatively independently of ethylene but acts partly through ethylene to suppress the transition from infection thread to nodule initiation.

Although nodule number was somewhat elevated in *na ein2* double mutants compared with *na* single mutants, the nodules that formed were still small and white (Foo *et al.*, 2016*b*). To determine if GA and ethylene may interact in mature nodules, we examined the expression of key gene expression markers in roots bearing nodules from *ein2*, *na ein2*, and wild-type plants (Supplementary Fig. S7). There was no significant difference between *ein2* and wild-type plants in the expression of any of the genes examined, suggesting that, once formed, ethylene-insensitive nodules are similar to wild-type nodules. In contrast, the expression of many of the genes was significantly different in *na ein2* nodules compared with wild-type or *ein2* nodules. Indeed, several patterns of gene expression observed in *na* nodules (Supplementary Fig. S6) were also seen in *na ein2*; the expression of *ENOD40* and *YC* was significantly reduced and the expression of *NIN* was significantly elevated in *na ein2* nodules compared with wild-type or *ein2* nodules (Supplementary Fig. S7). However, the expression of three genes, *ERF*, *YA1*, and *VAP*, showed differences in *na ein2* not observed in *na* nodules.

### Gibberellins influence nodule development and ultimate function

While the studies outlined above indicate key roles for GA in controlling both infection thread formation and nodule organogenesis, to date no studies have examined the influence of GA and/or DELLA signalling on the ultimate function of nodules, and in particular nitrogen fixation. We estimated nitrogen fixation using the acetylene reductase assay across various genotypes that were GA deficient, DELLA deficient, and/or both (note: ethylene evolution is expressed per g FW of nodules to account for differences in nodule number and size between genotypes). As GA-deficient *na* mutants form very few, and sometimes no, nodules, we employed two independent genetic approaches to elevate nodule number in the *na* background to a level that would allow us to perform acetylene reductase assays and compare the results with wild-type nodules. The lines assessed were the *na ein2* double mutant line (outlined above) and also *na nark* (also known as *na sym29*). The *na nark* double mutant line (Ferguson *et al.*, 2011) is GA deficient and also disrupted in *PsNARK*, a key element of the autoregulation of the nodulation pathway (Krusell *et al.*, 2002). Mutations in PsNARK lead to hypernodulation in both wild-type and GA-deficient *na* backgrounds, although, as previously reported, the nodules that form on *na nark* are smaller than those formed on the wild type or *nark* mutants and, like *na* single mutants, are small and white (Ferguson *et al.*, 2011).

**Fig. 7.**
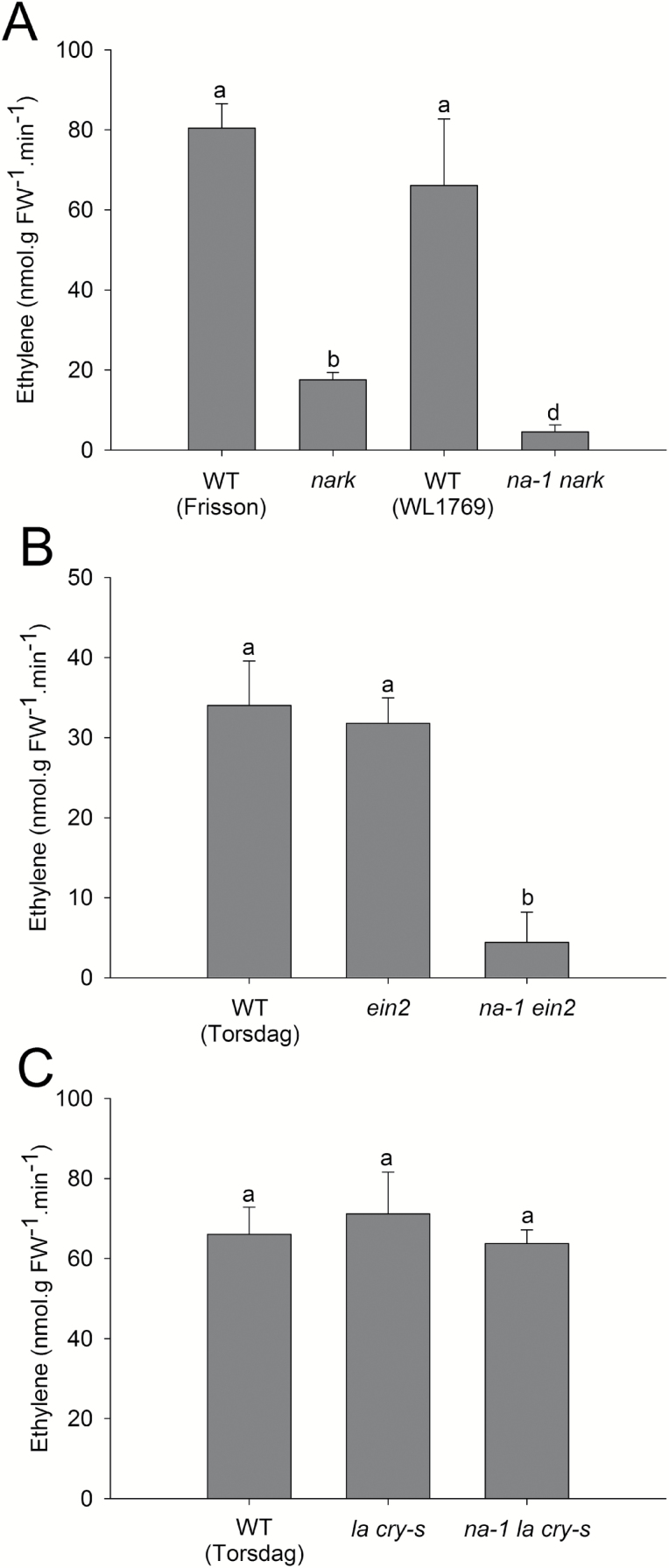
Acetylene reductase assay as an estimate of the ability of gibberellin-deficient lines carrying the *na-1* mutation to fix nitrogen compared with parental lines. Ethylene evolution per gram of nodule fresh weight (FW) from the acetylene reductase assay in (A) Wild-type (WT) lines (cv. Frisson and WL1769), the supernodulating autotoregulation mutant *nark*, and the double mutant *na-1 nark* plants. (B) WT (cv. Torsdag), ethylene-insensitive mutant *ein2*, and double mutant *na-1 ein2* plants. (C) WT (cv. Torsdag), DELLA protein mutant *la cry-s*, and triple mutant *na-1 la cry-s* plants. Plants were 4–5 weeks old and were inoculated when 7 d old. Values are mean ±SE (*n*=4). Values with different letters are significantly different (*P*<0.05).

As *nark* and *na* mutants have different wild-type progenitors, Frission and WL1769, respectively, we examined the acetylene reductase rate in both wild-type progenitors and found it was not significantly different (Fig. 7A). As previously reported, the acetylene reductase rate was reduced in *nark* mutants compared with wild-type lines (Cazenave *et al.*, 2014). The acetylene reductase rate was also significantly reduced in *na nark* double mutant nodules compared with either wild-type or *nark* nodules (Fig. 7A). *ein2* mutants had similar acetylene reductase activity to wild-type plants, and double mutant *na ein2* plants had significantly reduced acetylene reductase activity compared with *ein2* or wild-type lines (Fig. 7B). Therefore, GA-deficient plants in two independent genetic backgrounds displayed a significant reduction in acetylene reductase rate compared with wild-type plants. Indeed, the acetylene reductase rates in *na nark* and *na ein2* were ~14–25% of that observed in the *nark* or *ein2* parent, respectively. This suggests that nodules require GA to enable them to mature fully and reach normal wild-type levels of nitrogen fixation. This reduction in nitrogen fixation by GA-deficient nodules may be due to failure of the bacteria to differentiate into fully mature bacteroids and/or the premature deterioration of the bacteria, as discussed previously (Fig. 3).

The studies outlined above and previous reports have both demonstrated the influence of *della* mutations on early nodulation events and nodule number (Maekawa *et al.*, 2009; Ferguson *et al.*, 2011; Fonouni-Farde *et al.*, 2016; Jin *et al.*, 2016), but there has been no examination of the influence of *della* mutations on nodule function. We examined the acetylene reductase rate in DELLA-deficient *la cry-s* double mutants and in *na la cry-s* triple mutants. We found that although *la cry-s* double mutants produce fewer nodules than the wild type, the acetylene reductase rate of these nodules is not significantly different from nodules on wild-type plants (Fig. 7C). This suggests that constitutive GA signalling does not compromise nodule function, and is consistent with GA having a positive role in nodule function. Indeed, the acetylene reductase rate was also not significantly different in *na la cry-s* triple mutants compared with the wild type or *la cry-s* double mutants, indicating that the effect of *na* on nodule function is entirely mediated through the DELLA proteins.

## Discussion

In this study we demonstrate that GA plays a complex, dual role in the control of rhizobial infection and nodule development. GA, acting through DELLA proteins, suppresses infection thread formation. However, GA also acts through DELLAs to promote nodule organogenesis and therefore nodule function, a role only revealed by employing the severely GA-deficient mutants available in pea. This dual role is striking and may reflect the spatial separation of these events, as infection threads form in the root hairs of the epidermis and nodule organogenesis originates in the inner cortex. The clear developmental defects in the few nodules that do develop in GA-deficient plants, including their small size, relatively undifferentiated, damaged bacteroids, and their reduced nitrogen fixation rate, also indicate an important role for GA in promoting nodule maturation into nitrogen-fixing organs. Consistent with this action, *della*-deficient plants that display elevated GA signalling develop nodules that are of normal size, with wild-type levels of gene expression and function, clear evidence that GA does not suppress nodule organogenesis or ultimate function. Indeed, all of the phenotypes caused by GA deficiency in *na* mutants were rescued when combined with *della* mutant lines, the strongest evidence that GA acts through DELLAs to influence infection thread formation negatively and to promote nodule development positively.

**Fig. 8.**
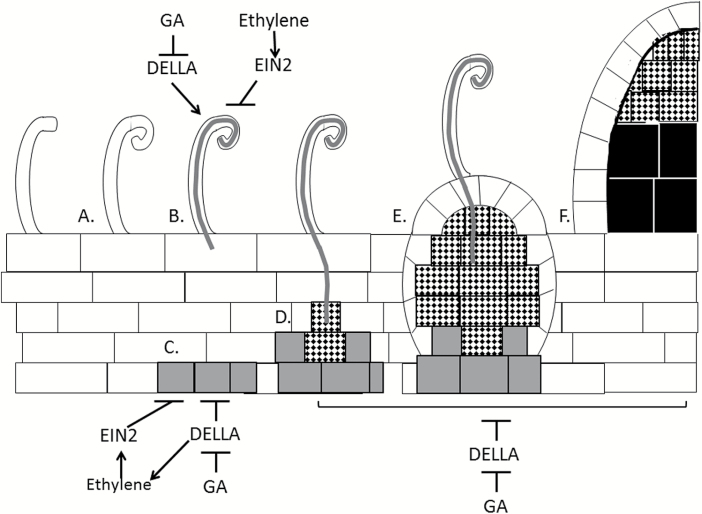
Model of the role of gibberellin (GA) and ethylene during nodule development in pea. The scheme shows infection and nodule development, including (A) root hair curling, (B) infection thread development, (C) initiation of cell division in the inner cortex (grey cells), (D, E) nodule organogenesis, including entry of bacteria (speckled cells), and (F) a mature nodule with cells containing enlarged nitrogen-fixing bacteroids (black cells). GA suppresses the action of DELLA proteins, and DELLA in turn promotes stage B and suppresses stages C–F. Ethylene acts through EIN2 to inhibit stages B and C. GA appears to act relatively independently of ethylene during stage B but can interact with ethylene at stage C.

The genetic studies presented here that demonstrate a negative role for GAs in suppressing infection thread development in pea (Figs 1, 4, 6) are entirely consistent with evidence from studies in a range of other legumes including *Lotus*, *Medicago*, and, during root hair entry, in *Sesbania* (Lievens *et al.*, 2005; Maekawa *et al.*, 2009; Ferguson *et al.*, 2011; Fonouni-Farde *et al.*, 2016; Jin *et al.*, 2016). For example, overexpression of dominant active MtDELLA1 protein elevated infection thread formation in *Medicago* but did not increase nodule number (Fonouni-Farde *et al.*, 2016). In pea, this effect on the number of infection threads does not appear to be due to an influence of GA on root hair number, length, or curling in response to bacterial challenge (Supplementary Fig. S1; data not shown). Furthermore, the reduction in infection threads seen in *della*-deficient pea lines, including those on a GA-deficient background, is entirely consistent with the reduced number of nodules observed in these lines (Fig. 4; Ferguson *et al.*, 2011). What is striking, however, is that GA-deficient *na* mutants display both strongly elevated numbers of infection threads (more than five times those in the wild type) and strongly reduced number of nodules, and often no nodules at all (Fig. 1). This clearly illustrates that in addition to suppressing infection thread formation at the epidermis, GA is also required to promote nodule organogenesis in the cortex (Fig. 8).

Until now we have had little information on the influence of GA on nodule organogenesis, development, or ultimate function. By examining the effect of low GA levels, caused by the *na* mutation, and elevated GA signalling, caused by DELLA deficiency, on nodule development and function we have clearly defined a positive role for GA in these later stages of nodulation. It is vital to note that the nodules that do form on *della*-deficient mutants did not display altered development or function. Nodules on *della*-deficient *la cry-s* mutants are similar in size to wild-type nodules (Ferguson *et al.*, 2011), have a similar ability to fix nitrogen (Fig. 7), and display no change in the expression of a range of nodule-associated genes (Supplementary Fig. S6). This is significant, as it indicates that elevated GA signalling does not preclude normal nodule development. Indeed, the fact that the few nodules that do develop on GA-deficient *na* mutants are considerably smaller than wild-type nodules, and application studies with GA_3_ or the GA biosynthesis inhibitor PAC demonstrate that nodule size is promoted by GA (Fig. 5A; Supplementary Table S2), is consistent with the hypothesis that GA promotes nodule organogenesis and development. This positive effect of GA on nodule size is also found in lupin (Fig. 5B), a legume species that does not use root hair entry of infection threads, confirming that GA can influence nodulation independently of infection thread development.

The positive influence of GA on nodule development has consequences for the bacteria hosted inside the maturing nodule. An examination of bacteria inside nodules of the wild type and GA-deficient *na* mutants revealed a striking difference between genotypes in the size and integrity of bacteroids in the central zone of the nodule. Unlike the large bacteroids in wild-type nodules, bacteria inside nodule cells of *na* mutants were small and more closely resembled bacteria inside infection threads (Fig. 3). Indeed, these bacteria inside *na* nodules appeared to be undergoing premature senescence, reflected by the observed damage to the peribacteroid and symbiosome membranes. These physical defects in nodule maturation, including the bacteroids, are the likely cause of the reduction in nitrogen fixation rate displayed by GA-deficient nodules (Fig. 7). Consistent with the defective nodule development observed in *na* plants is the altered expression of some nodule marker genes found in GA-deficient *na* nodules compared with wild-type nodules (Supplementary Fig. S5).

Studies using the GA-deficient *na* mutant have also revealed the intriguing phenomenon of bacterial accumulations. A small proportion of the infection threads that form in *na* mutants grow down into the root cortex where they ramify but are not accompanied by any cortical cell division associated with nodule development (Figs 2, 3; Supplementary Fig. S2). The plant cell wall and membrane surrounding these structures and the bacteria inside these accumulations are similar in size and appearance to those in infection threads (Fig. 3C, F). Bacterial accumulations were also observed, but only rarely, in ethylene-insensitive *ein2* mutants and in wild-type plants treated with the GA biosynthesis inhibitor, PAC. These unique structures do not appear to be similar to any infection thread-related structures reported for symbiotic mutants. One parallel may be the broad and irregular infection threads observed in *Sesbania* plants treated with a GA biosynthesis inhibitor (Lievens *et al.*, 2005). The formation of these structures in GA-deficient roots may support a role for GA in suppressing infection thread development, aiding the entry of the bacteria into the host’s cells and/or the need for GA to promote the cell division required for normal nodule development.

The influence of elevated GA and/or DELLA levels on the induction of early nodulation gene expression in the hours following treatment with Nod factors has been well studied and indicates an overall negative effect of GA signalling, through DELLAs, on these responses (Fonouni-Farde *et al.*, 2016; Jin *et al.*, 2016). We examined the expression of some of these early nodulation markers in the GA-deficient *na* mutant in the days following challenge with bacteria (Supplementary Fig. S3). Given the phenotype of *na* mutants, which combines increased infection threads with reduced nodule development at the whole-root level, it is not surprising that we found both elevated and reduced expression of these genes at different time points after inoculation. Furthermore, it is interesting to note that gene expression studies in several species indicate that GA biosynthesis may be up-regulated during interaction with rhizobia. Reports in soybean and *Sesbania* indicate altered expression of key GA biosynthesis and catabolism genes during the hours and days following inoculation and in some cases within the developing nodule itself, suggesting that bioactive GA levels may be elevated (Lievens *et al.*, 2005; Libault *et al.*, 2010; Hayashi *et al.*, 2012). These changes in GA biosynthesis/catabolism gene expression appear to require Nod factors, as they are not induced when plants are challenged with Nod factor-deficient rhizobia (Lievens *et al.*, 2005; Hayashi *et al.*, 2012). However, GA levels in inoculated roots and/or nodules have not been quantified using modern analytical techniques with labelled standards and, given the well-known feedback regulation of the expression of genes in the GA pathway (Elliott *et al.*, 2001), it is not clear whether bioactive GA levels are indeed elevated during nodulation. We therefore examined the expression of key GA metabolism genes and determined the level of GA_1_ during infection, and found no overall change in the expression of the GA genes or GA_1_ content of roots in the hours and days following rhizobial challenge (Supplementary Fig. S4). However, it is possible that the GA content is spatially regulated at a fine scale during infection and hence differences may not be apparent when whole sections of root are analysed. Indeed, when gene expression was examined in isolated root hairs of *Medicago* challenged with Nod factors, elevated expression of genes coding for GA biosynthesis genes and suppression of expression of GA receptor genes was observed (Breakspear *et al.*, 2014).

Previous studies in pea have revealed an interaction between ethylene and GA during nodulation. The GA-deficient *na* mutants produce more ethylene, and this appears to contribute to the low number of nodules in this mutant as nodule number can be partially restored by combining *na* with the ethylene-insensitive *ein2* mutant or treatment with an ethylene synthesis inhibitor (Ferguson *et al.*, 2011; Foo *et al.*, 2016*b*; Fig. 6). More detailed examination of this interaction using *na ein2* double mutant plants and their respective parental lines revealed that ethylene and GA appear to act relatively independently during infection thread development, but GA appears to interact with ethylene to promote nodule initiation (Fig. 8), since infection thread numbers are still high in *na-1 ein2* mutant plants, but these double mutants form significantly more nodules than *na-1* parents (Fig. 6). However, GA appears to act independently of ethylene in promoting nodule development and ultimate function, as nodules that form on *na ein2* mutants are of similar size to those on *na* plants (Foo *et al.*, 2016*b*) and display reduced nitrogen fixation (Fig. 7).

This study clearly validates the theory of a dual role for GA during infection thread formation and nodule development (Fig. 8). This is consistent with the spatial separation of the epidermal and cortical programmes during nodulation (Oldroyd and Downie, 2008) and was only revealed by examining the influence of both elevated and reduced GA levels and signalling. Given that it is still not entirely clear how and through which signals the epidermal and cortical programmes are co-ordinated (Oldroyd, 2013), this dual role for GA might offer insights into this complex process. It was recently revealed that some rhizobia can produce GA, although this is not the case for bacteria that inhabit indeterminate nodules, such as those formed on pea by *R. leguminosarum* (Hershey *et al.*, 2014; Tatsukami and Ueda, 2016; Nett *et al.*, 2017). It will be interesting to consider this dual role in the context of an increased understanding of GA biosynthesis in at least some rhizobia that lead to nodulation, such as those that form determinate nodules in *Lotus* and soybean (e.g. Tatsukami and Ueda, 2016; Nett *et al.*, 2017). Future studies could examine whether there is spatial regulation of endogenous GA levels and/or responses during infection and nodule development. Furthermore, as previous studies have examined the interactions of DELLA proteins with downstream elements of the Nod factor response *in vitro* (Fonouni-Farde *et al.*, 2016; Jin *et al.*, 2016), future studies should examine whether *in planta* DELLA interactions are under spatial or temporal control during infection and nodule development.

## Supplementary data

Supplementary data are available at *JXB* online.

Fig. S1. Root hair curling in wild-type (WT) and *na-1* after inoculation with *Rhizobium leguminosarum* bv. *viciae*.

Fig. S2. A cross-section of a *na* root containing a bacterial accumulation (white arrows), nuclei (n), outer cortex (oc), inner cortex (ic), endodermis (ed), and vascular bundle (vb) 5 weeks after inoculation with *Rhizobium leguminosarum* bv. *viciae*.

Fig. S3. The expression of early nodulation (*ENOD*) genes and *NIN* in wild-type (WT) and *na-1* plants 0, 2, and 4 d following inoculation with *Rhizobium leguminosarum* bv. *viciae*.

Fig. S4. Gibberellin (GA) levels and expression of GA metabolism genes in root tips of wild-type (WT) and symbiosis mutant *dmi2* plants after inoculation with *Rhizobium leguminosarum* bv. *viciae*.

Fig. S5. Relative expression of nodule marker genes in wild-type (WT) and *na-1* roots bearing nodules after inoculation with *Rhizobium leguminosarum* bv. *viciae*.

Fig. S6. Relative expression of nodule marker genes in wild-type (WT), double mutant *la cry-s* (*della*), and triple mutant *la cry-s na-1* (*na della*) roots bearing nodules after inoculation with *Rhizobium leguminosarum* bv. *viciae*.

Fig. S7. Relative expression of nodule marker genes in wild-type (WT), *ein2*, and double mutant *na-1 ein2* roots bearing nodules after inoculation with *Rhizobium leguminosarum* bv. *viciae*.

Table S1. Primer pairs used in this study.

Table S2. Nodule number per g DW of root and average individual nodule size (DW mg) in wild-type plants treated with various doses of the gibberellin biosynthesis inhibitor, paclobutrazol (PAC) 4 weeks after inoculation with *Rhizobium leguminosarum* bv. *viciae*.

Supplementary Figures and Tables

## Author Contributions

ELM and EF performed the experiments and analysed the data; EF conceived the project and wrote the article with contributions from JBR and ELM.
