# Supplementary material for: Gibberellins promote nodule organogenesis but inhibit the infection stages of nodulation
Source: J Exp Bot. 2018 Feb 8;69(8):2117–30. doi: 10.1093/jxb/ery046 (PMC6018947; doi:10.1093/jxb/ery046)
Supplement: Supplementary Figures and Tables [file ery046_suppl_supplementary_figures_and_tables.pdf]

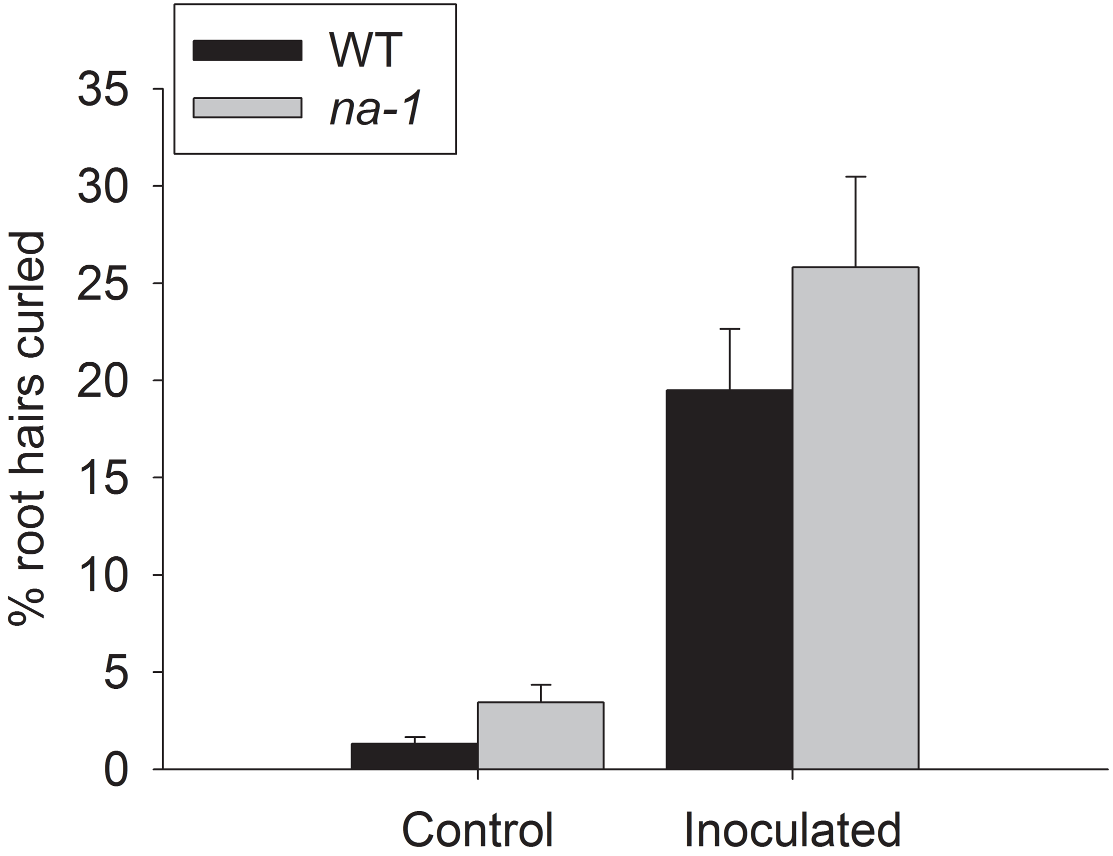

Supplementary Figure 1. Root hair curling in wild type (WT) and *na-1* after inoculation with *Rhizobium leguminosarum* bv. *viciae*. Percentage of root hairs curled 10 d after treatment with rhizobium (inoculated) compared with a solvent control (control) (n=12-16) and analysis by ANOVA indicated no significant differences between *na-1* and its WT progenitor.

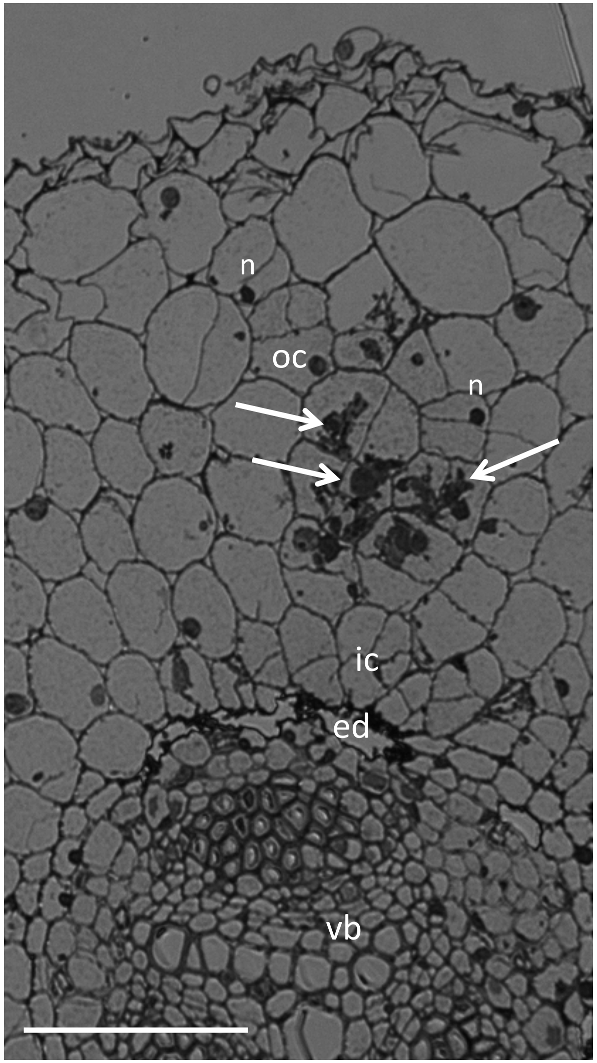

Supplementary Figure 2. A cross section of a *na* root containing a bacterial accumulation (white arrows), nuclei (n), outer cortex (oc), inner cortex (ic), endodermis (ed) and vascular bundle (vb) 5 weeks after inoculation with *Rhizobium leguminosarum* bv. *viciae*. Scale bar is 100 µm.

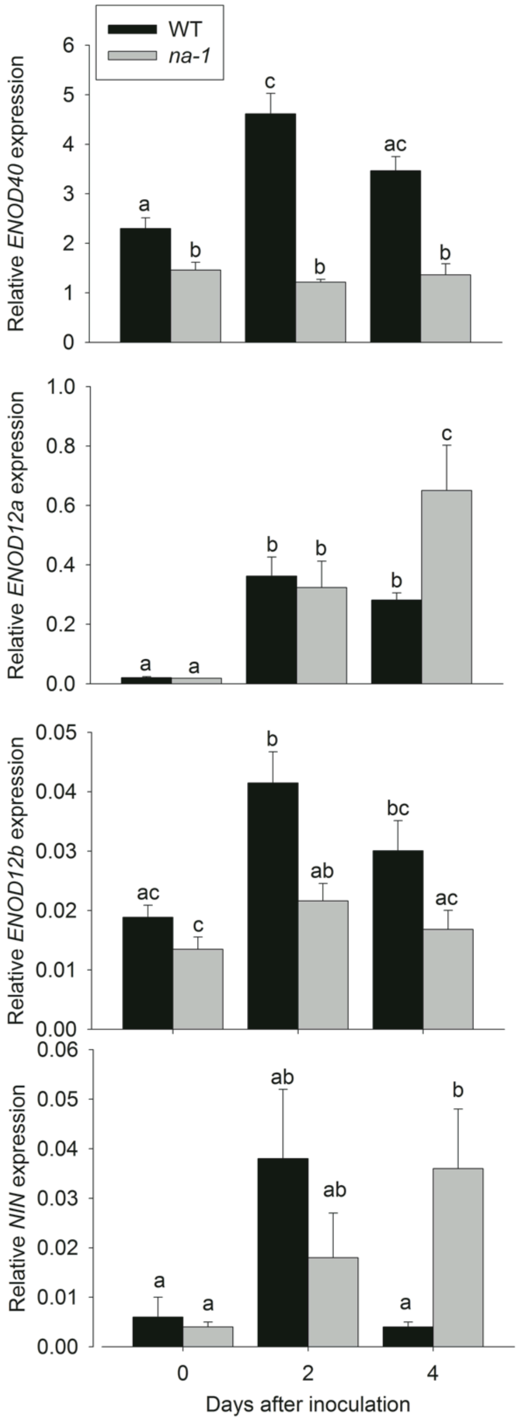

Supplemental Figure 3. The expression of early nodulation (*ENOD*) genes and *NIN* in wild type (WT) and *na-1* plants 0, 2 and 4 d following inoculation with *Rhizobium leguminosarum* bv. *viciae*.
Relative expression of *ENOD40*, *ENOD12a*, *ENOD12b* and *NIN*. Values are mean ± s.e. (n=3-4). Values with different letters are significantly different ($P<0.05$).

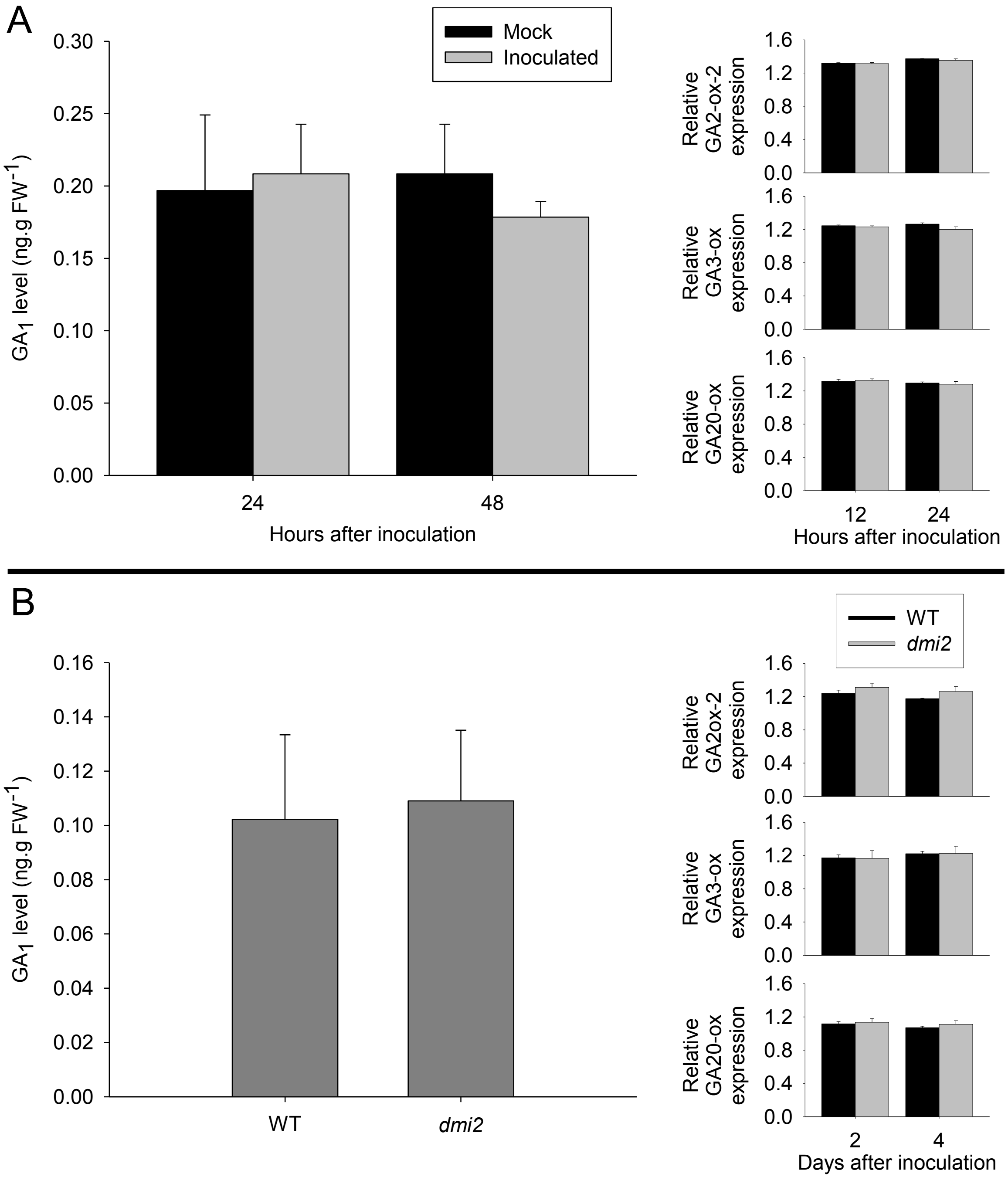

Supplementary Figure 4. Gibberellin (GA) levels and expression of GA metabolism genes in root tips of wild type (WT) and symbiosis mutant *dmi2* plants after inoculation with *Rhizobium leguminosarum* bv. *viciae*.

(A) Levels of the bioactive GA_1 and relative expression of GA genes *GA2-ox-2, GA3-ox* and *GA20-ox* in root tips of WT pea plants in hours and days after treatment (inoculated) or with a solvent control (mock) (n=3-5). (B) WT and *dmi2* mutants, GA_1 in root tip (n=4) 12 d after inoculation and relative expression of GA genes 2 and 4 d after inoculation (n=3). Values are mean ± s.e. and analysis of each experiment by ANOVA or t-test indicated no significant differences.

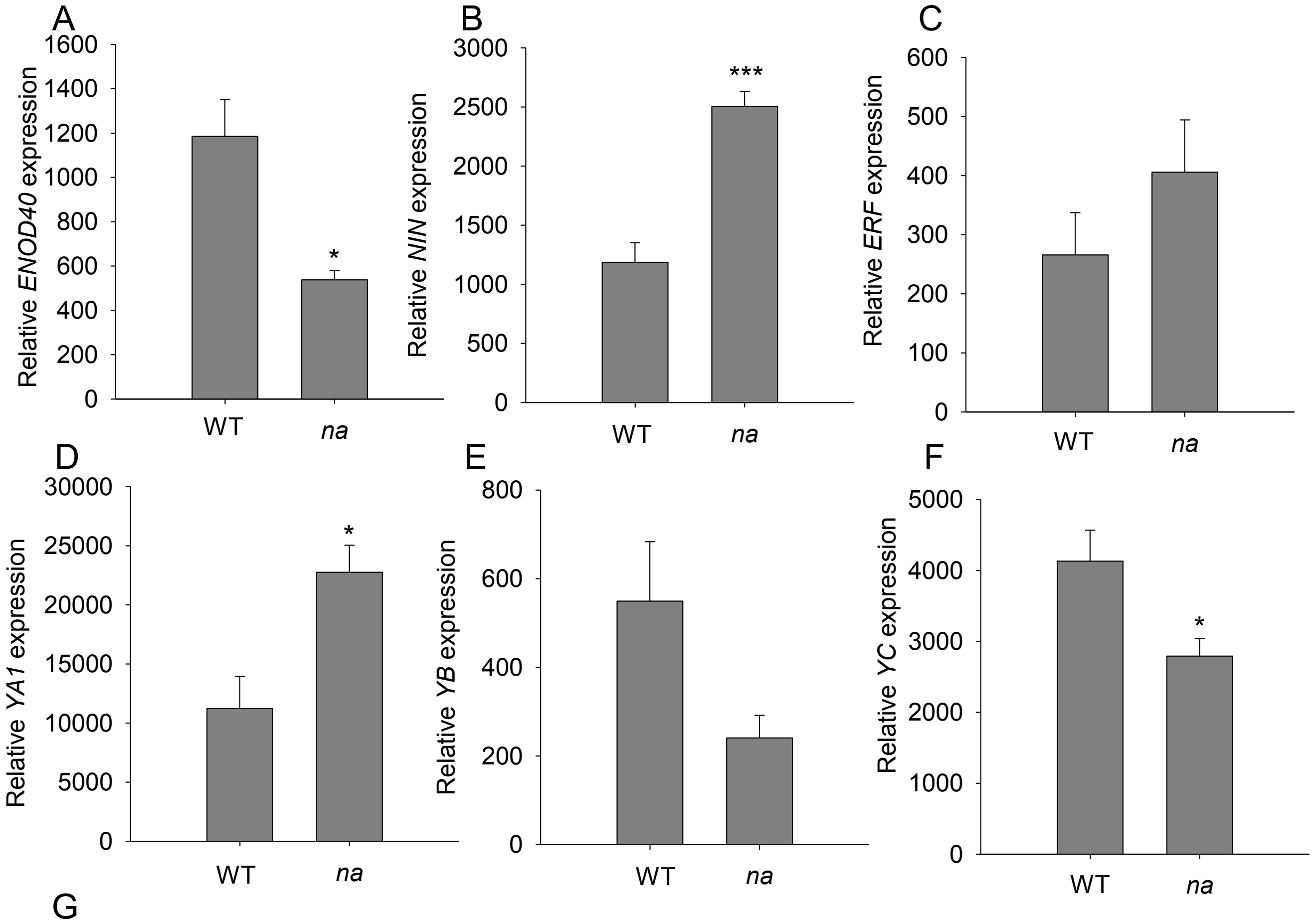

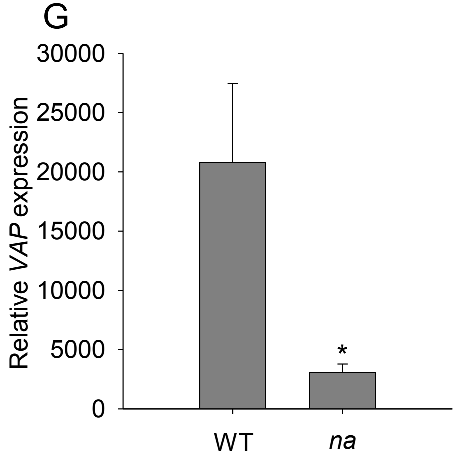

Supplementary Figure 5. Relative expression of nodule marker genes in wild type (WT) and *na-1* roots bearing nodules after inoculation with *Rhizobium leguminosarum* bv. *viciae*.

Nodules with small amount of root tissue were harvested from 35 d old plants, 28 d after inoculation with *Rhizobium leguminosarum* bv. *viciae*. Relative expression of nodule marker genes *ENOD40*, *NIN*, *ERN*, *YA1*, *YB*, *YC* and *VAP*. Values are mean ± s.e. (n=3-4), asterisks indicate significant difference from WT (*P<0.05, ***P<0.001).

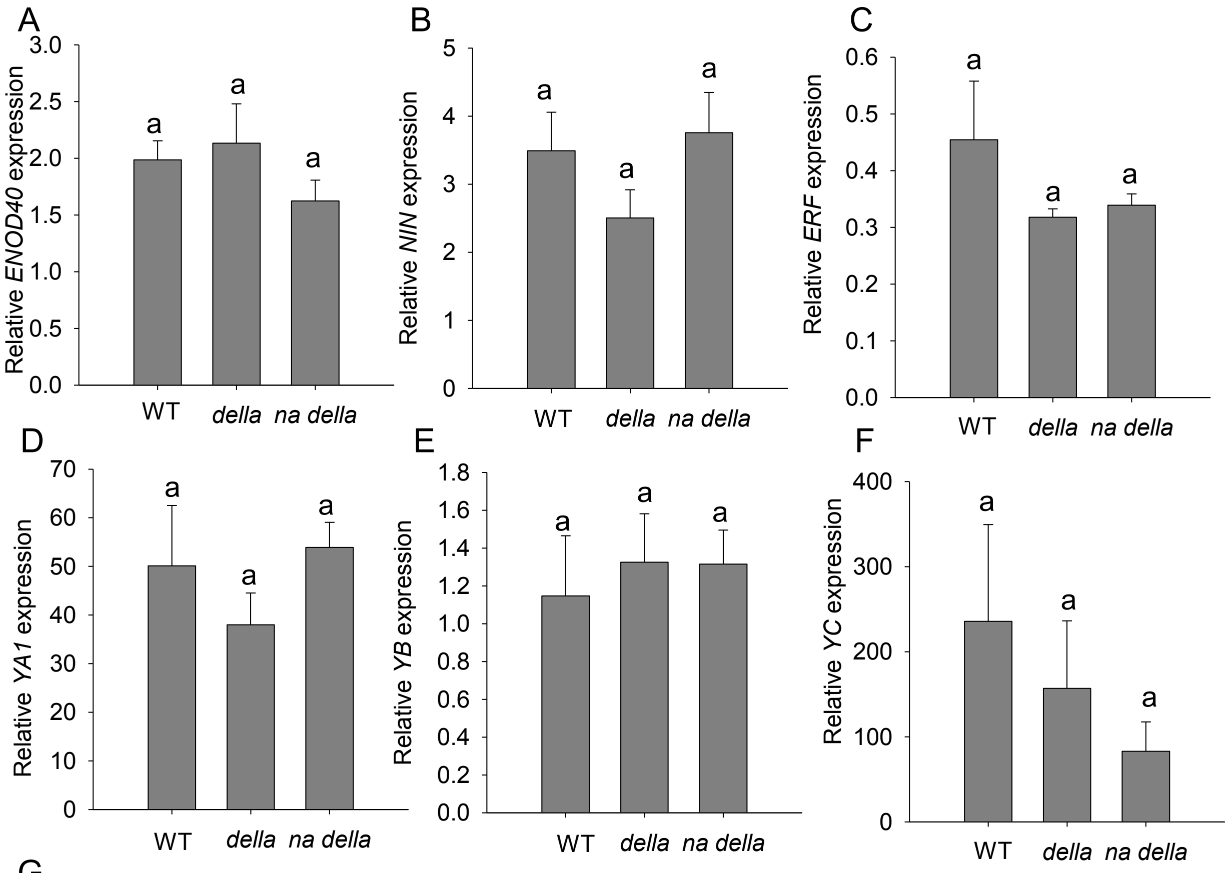

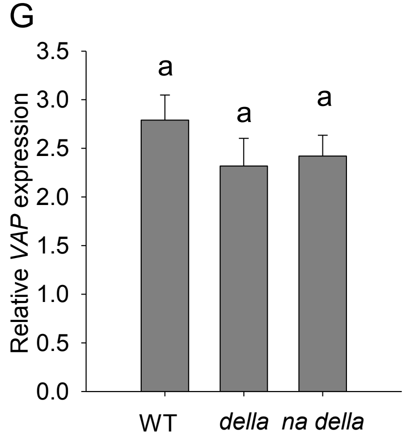

Supplementary Figure 6. Relative expression of nodule marker genes in wild type (WT), double mutant *la cry-s* (*della*) and triple mutant *la cry-s na-1* (*na della*) roots bearing nodules after inoculation with *Rhizobium leguminosarum* bv. *viciae*.
Nodules with small amount of root tissue were harvested from 35 d old plants, 28 d after inoculation with *Rhizobium leguminosarum* bv. *viciae*. Relative expression of nodule marker genes *ENOD40*, *NIN*, *ERN*, *YA1*, *YB*, *YC* and *VAP*. Values are mean ± s.e. (n=3-4), ANOVAs were performed and no significant differences were found.

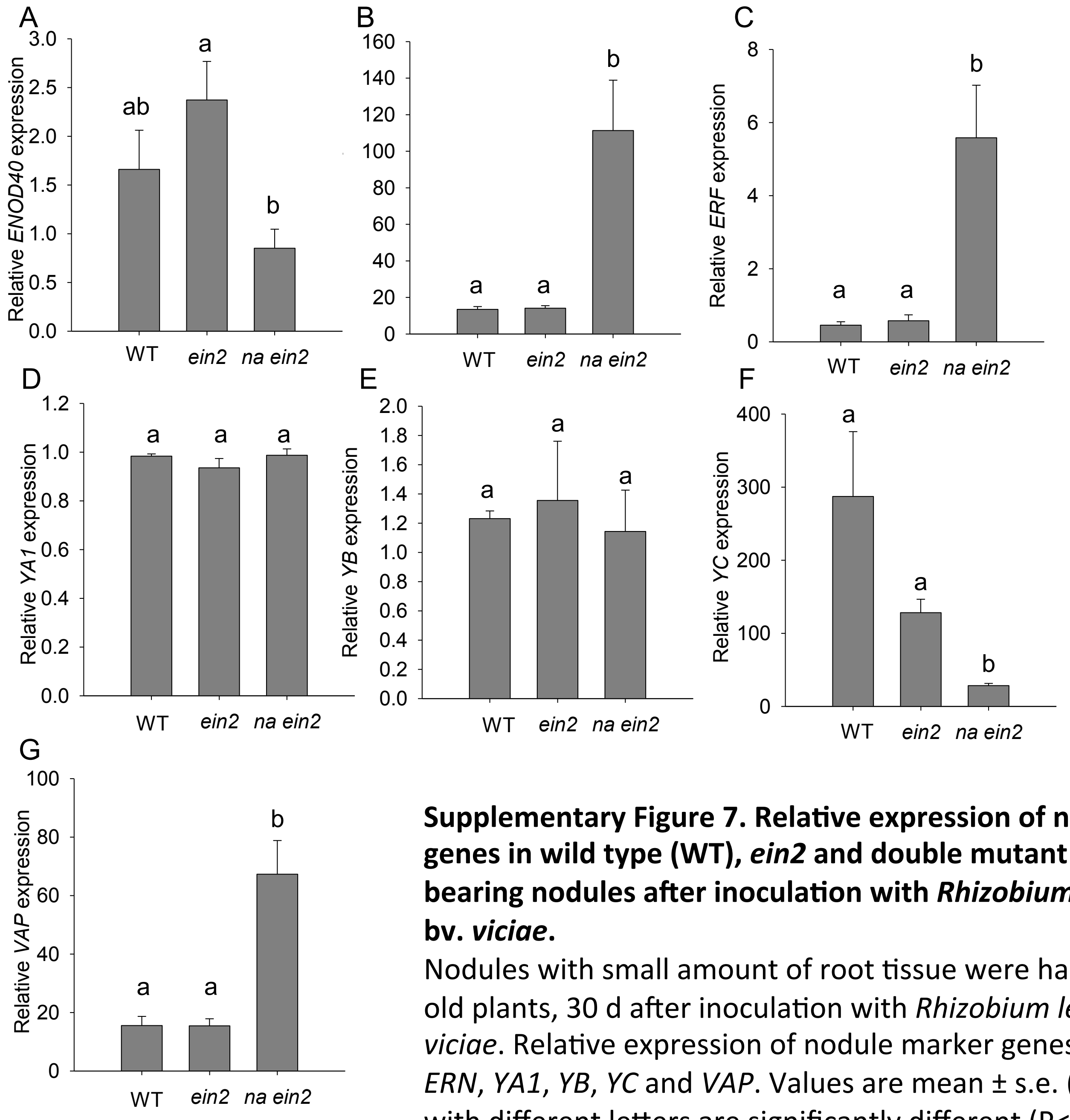

Supplementary Figure 7. Relative expression of nodule marker genes in wild type (WT), *ein2* and double mutant *na-1 ein2* roots bearing nodules after inoculation with *Rhizobium leguminosarum* bv. *viciae*.

Nodules with small amount of root tissue were harvested from 37 d old plants, 30 d after inoculation with *Rhizobium leguminosarum* bv. *viciae*. Relative expression of nodule marker genes *ENOD40*, *NIN*, *ERN*, *YA1*, *YB*, *YC* and *VAP*. Values are mean ± s.e. (n=3-4), values with different letters are significantly different (P<0.05).

Supplementary Table 1. Primer pairs used in this study.

Gene name	F primer	R primer
PsERN	5’-TCTCTTTCGAGCGAGGCTAC-3’	5’-AGCTAGACGCGTCGATCATT-3’
PsENOD12b	5’-TGAACCACCAGTGAATGAGC-3’	5’-TGGATGTTATGTTCCGCTGT-3’
PsYA1	5’-ATGTCGGTTGATGCACAAAA-3’	5’-ACGAAGTTGGTCCGTCGTC-3’
PsYB	5’-GGCATTAAGGAGCAAGATCG-3’	5’-TGCCTCACTTGTCACAAAGC-3’
PsYC	5’-GTGGAGGTCAAATGCCGTAT-3’	5’-TGGACTGTTGCTGAAGTTG-3’
PsNIN	5’-GGTGGTGGATGCAGTGTTTC-3’	5’-GAATGCTGTAATGTCGATTGCG-3’
PsVAP	5’- CTCCAGGTGCAGCTATCAAA-3’	5’-CAAGCACTTCTCTTATGTCATCCA-3’
PsTF11a	5’-TCTTCCCGTCCTTCCACATAA-3’	5’- GCAACCTCCTTCTCCTTGGAT-3’

Supplementary Table 2. Nodule number per g DW root and average individual nodule size (DW mg) in wild type plants treated with various doses of the gibberellin biosynthesis inhibitor, paclobutrazol (PAC) 4 weeks after inoculation with *Rhizobium leguminosarum* bv. *viciae*. Values are mean ± s.e. (n=5-6) and values with different letters are significantly different ($P<0.05$), n.m. is not measurable.

Treatment	Nodule number per g root DW	Individual nodule DW
Control	1022.2 ± 98.7 a	0.0865 ± 0.0110 a
+ 1 µg PAC	928.5 ± 129.6 a	0.0998 ± 0.0129 a
+ 3 µg PAC	1161.9 ± 224.4 a	0.1100 ± 0.0392 a
+ 10 µg PAC	1692.2 ± 267.6 a	0.0349 ± 0.0119 b
+ 30 µg PAC	589.4 ± 65.8 b	n.m.
